# Twenty steps to ingrain power asymmetry in global health biomedical research

**DOI:** 10.1371/journal.pbio.3001411

**Published:** 2021-09-30

**Authors:** Iruka N. Okeke

**Affiliations:** Department of Pharmaceutical Microbiology, Faculty of Pharmacy, University of Ibadan, Oyo State, Nigeria

## Abstract

Health research in low‐income settings must prioritize sustainability in order to truly impact target diseases in the long term; this Perspective article satirically summarizes how biomedical investigators from high-income countries can collaboratively work to (not) accomplish this.

## Teaser

Health research in low-income settings must prioritize sustainability to truly impact target diseases in the long term. Here, I satirically summarize how biomedical investigators from high-income countries can collaboratively work to (not) accomplish this.

Are you a laboratory scientist par excellence with international research ambitions? You could address scientific roadblocks in the important and highly fundable field of global health, which is rich in “partnerships” but poor in true technology transfer [1,2] by closely, or even loosely, following these 20 steps. As your interest is in “global health” and my tip list draws on common stereotypes, I’ll presume that you are a high-income country scientist with low-income country research aspirations.

1. Solitarily develop your hypotheses, aims, work plan, experimental setup, and timeline before you have decided who to collaborate with or where the research will take place.
2. Pick a Partner Country. Avoid locations where good research in the area has previously been conducted. Instead, prioritize countries with pristine beaches, a game reserve, or some other “must see.” Holiday brochures could be helpful at this stage.
3. As one of your aims will be to build capacity, avoid brainstorming with anyone with overlapping or complementary expertise. Seek collaborator recommendations from your institution’s foreign students’ association, or ask someone who resembles people in your holiday brochure. You may come across information that women in your Partner Country could be diverted from research by community pressure to prioritize child-rearing. To avoid this type of complication, pick a male partner, and if his speciality is different, convince him that your area is more important. Once you have his name, submit your local institutional review board (IRB) proposal quickly, before external parties attempt to poke holes in your protocol. Avoid time-wasting conversations with ethicists, social scientists, and statisticians without the chemistry and biology foundation necessary to usefully contribute to your work. Should any of them hem you into a meeting, emphasize that your primary motivation is decolonizing global health.
4. If you did your planning right, you will have selected methods perfected in your institution’s state-of-the art lab. Your protocol should differ from any existing consensus guidelines for working in resource-limited settings so that you can be credited for originality.
5. Don’t engage with any Partner Country vendors. Buy equipment and consumables from home country dealers you know and trust, and transport them in your checked luggage. If the holiday brochure mentions a different electricity rating in Partner Country, grab a portable electric power converter at a duty-free shop on your way out.
6. Ahead of your arrival, instruct your Partner Country contact to get local ethical approval for the research you’ve designed. Discussions at this stage should focus on how to get the proposal through the local ethics committee in time for your visit and not its content.
7. Your research visit should take place during your spring break, irrespective of whether this is peak infection time for the disease of interest. When you arrive in your Partner Country (this is called being “in-country”), ask to be introduced to a traditional ruler and take selfies with local children outside the palace gates. This is community engagement. Don’t spend time seeking audience with Ministry of Health officials, healthcare practitioners, patient advocacy groups, or scientists other than your partner-designate. They are busy dealing with the type of bureaucracy that could slow you down and unlikely to understand what you are doing or why it is important.
8. If you need skilled bench or field assistants, hire them away from complementary in-country programs by offering monetarily higher but short-term contracts. Encourage any enrolled in educational or career development programs to disengage and focus on your innovative project, which is structured so that they cannot enroll in any programs at your home institution.
9. Should you find that your host lab is not configured to use your materials, rapidly adapt and monogram a porta-cabin or tent and power equipment with a portable generator or via your laptop’s USB. You can then label the workspace “my field lab,” even if it is located within an inner city research institute and evade local biosafety oversight, allowing you to store toxic experimental waste in a corner.
10. Your in-country partner has no relevant expertise; this is why you brought the research. Train him and his students on the assay you developed so that they can do the monotonous testing after you depart. Structure training as a short workshop with good food, generous per diems, and inkjet-printed certificates. Focus on your standard operating procedure (SOP), and do not be diverted by participant questions about troubleshooting, comparable assays, interpretations, or procurement.
11. Set up your Partner Country personnel (henceforth referred to as “your Guys”) to enter project data on a tablet that automatically syncs to a computer server in your home institution [3]. Should your Guys request database access, explain that you lack authority to issue log-ins for your host institution’s server and that, with power outages and no systems administrator, an in-country server cannot be installed. For security reasons, the tablet should only be capable of running project-specific apps that you install.
12. On your second Partner Country visit, consider bringing along some trainees to help with your capacity building endeavor. Rather than highly skilled postgraduates from your group, choose students with little foundation, who will benefit maximally from the experience and whose absence from home will not affect your lab’s productivity. Do a crash course on the SOP with them on the plane, and get them to draft the introduction of a future publication they could lead. Once you arrive, offer a second in-country workshop on your SOP. If there are no more local students to train, bus in per diem enthusiasts from a proximal nonendemic state. Let your students run this course while you take memorable pictures for your institution’s global health flyers.
13. Because it is difficult for Partner Country nationals to secure visas, international conference presentations on the work done in “your field lab” should be presented by you or the trainee teaching assistants that ran the second workshop. After all, they have already written the introduction.
14. Should your Guys request more advanced training or a desire to perform follow-up experiments, explain why such high-tech techniques are not needed in-country. If the follow-up ideas are good, have the samples shipped to your lab so that the suggested experiments can form the basis of a home institution PhD project. Don’t bother to tell the student you recruit to this project where the ideas or samples came from or to acknowledge their source in outputs from the work. If your Guys persist in requests for inappropriately high-tech engagement, offer brochures from fee-charging postgraduate programs elsewhere. To avoid pesky consular complications (Step 13), don’t consider bringing them to your lab where you perform these techniques routinely.
15. If field lab progress is slower than anticipated, decline any no cost extensions your funder might offer. Wrap up the project as quickly as possible to minimize risk, trimming the objectives to what can realistically be accomplished on the difficult Partner Country terrain. During wrap-up, initiate proposals to replicate the work in your Next Favorite Country. In global health, your presence in disadvantaged countries on different continents should be prioritized.
16. A key to securing funding to enter more countries is publication. Local coauthorship could, like ethics approval and sequence database accession numbers, be demanded from global health journal editors. If your Guys are distracted by their day jobs or struggle with writing for your preferred journals because English is not their first language, generously offer to prepare manuscripts single-handedly. Don’t lose time composing policy documents, public health guidelines, or anything that cannot appear in a high-impact journal. Should you encounter writer’s block, draw on influential guides from other disciplines, which will resonate if you find these tips valuable [4,5].
17. Toward expanding footprint, take key and costly pieces of equipment from your original Partner Country for servicing and then reallocate them to your Next Favorite Country, where they will be more useful because of Partner Country reagent procurement difficulties. The tent sheltering hazardous waste should remain in Partner Country, so that you can continue to present your field lab as a regional center for excellence. Even though they have never designed a study, adapted a protocol, or communicated on the work with anyone but you, your Guys are on the verge of becoming leaders in the field, thanks to the capacity you built.
18. Never make the mistake of putting your Guys in Partner Country in contact with scientists in your Next Favorite Country. Keep communication lines simple and always routed through you. Under no circumstances should you share successful grant proposals or early paper drafts with any country Guys.
19. Should the idea your brilliant PhD student pursued (Step 14) show promise for diagnostic test development, use the student’s thesis as the basis for a patent and a start-up company. As you are more interested in helping people than making money, your priority should be to sell the company. Dispel investor fears about the absence of a viable endemic area market by suggesting that diagnostic companies focus on the small but much wealthier returning traveler market.
20. Build acknowledgment collages from training workshop photos, particularly those that included bused-in out-of-state trainees. Place these at the end of the increasing number of talks you will be invited to give. As there are so many faces in the collages, don’t clutter the slides with names.

By this point, I can almost guarantee that you will be internationally renowned, with a network spanning most of your disease’s expanding endemic reach. Expanding endemicity is an intractable problem, but also a research boon, in global health, a field rich in power imbalance and, invariably, satire [4–8].
